# Association of Genetically Predicted Activity of AMP Deaminase 1 with Clinical and Biochemical Parameters in Diabetic Individuals with Coronary Artery Disease

**DOI:** 10.3390/ijms26168071

**Published:** 2025-08-21

**Authors:** Maria Pietrzak-Nowacka, Ewa Gątarska, Krzysztof Safranow, Agnieszka Boroń, Kazimierz Ciechanowski, Jeremy S. C. Clark, Andrzej Ciechanowicz, Dorota Kostrzewa-Nowak

**Affiliations:** 1 Department of Nephrology, Transplantology and Internal Medicine, Pomeranian Medical University, 70-111 Szczecin, Poland; mariola.nowacka@o2.pl (M.P.-N.); ewga@op.pl (E.G.); kazimierz.ciechanowski@pum.edu.pl (K.C.); 2 Department of Biochemistry and Medical Chemistry, Pomeranian Medical University, 70-111 Szczecin, Poland; krzysztof.safranow@pum.edu.pl; 3 Department of Clinical and Molecular Biochemistry, Pomeranian Medical University, 70-111 Szczecin, Poland; agnieszka.boron@pum.edu.pl (A.B.); jeremy.clark@pum.edu.pl (J.S.C.C.); andrzej.ciechanowicz@pum.edu.pl (A.C.)

**Keywords:** type 2 diabetes, coronary artery disease, adenosine monophosphate deaminase 1, gene polymorphism

## Abstract

Some reports indicated the association of rs17602729 and rs34526199 functional polymorphisms of the *AMPD1* gene encoding adenosine monophosphate deaminase 1 (AMPD1) with the risk of coronary artery disease (CAD) and/or its intermediate phenotype. Therefore, the aim of our study was to analyze the association of both *AMPD1* polymorphisms with the predisposition to disease and both clinical and biochemical phenotypes but solely in diabetic individuals with CAD. The study group consisted of 196 adult diabetic individuals with CAD, and the control group comprised 200 healthy newborns. Both *AMPD1* polymorphisms were identified by a SNaPshot minisequencing reaction. Clinical and laboratory data were taken from patients’ records. There were no significant differences between both groups in the frequency distributions of *AMPD1*:rs17602729 and rs34526199 alleles or genotypes. BMI and the frequency of obesity in TT rs17602729 homozygotes (no AMPD1 activity) were significantly lower and the serum concentration of HDL cholesterol was significantly higher compared to other patients. The concentrations of total cholesterol and LDL cholesterol in homozygotes for wild-type *AMPD1*:rs17602729 (c.34C) and rs34526199 (c.860A) alleles (full AMPD1 activity) were significantly lower compared to its values in other patients. Our results suggest that genetically predicted activity of AMPD1 is associated with variation in body mass and lipid metabolism in diabetic Polish people with CAD.

## 1. Introduction

Adenosine monophosphate deaminases (AMPDs) catalyze the irreversible deamination of adenosine monophosphate (AMP) to inosine monophosphate (IMP) and play an important role in the purine nucleotide cycle [1]. AMP deaminase 1 (AMPD1), the skeletal muscle-specific AMPD isoform, encoded in humans by the *AMPD1* gene, is located at chromosome 1p13.2 [2]. The common c.34C>T transition (rs17602729, a single nucleotide polymorphism; SNP) in the *AMPD1* gene introduces a premature termination codon in exon 2 (p.Gln12Ter) and results in an inactive enzyme [3].

In 1999, Loh et al. published a report indicating that patients with heart failure (HF) who were carriers of the rs17602729:c.34T allele experienced significantly longer survival from the onset of HF symptoms to referral of heart transplantation compared to CC homozygous HF patients [4]. However, a meta-analysis by Feng et al. [5] based on the aforementioned report and seven later publications (including our study from 2009 [1]) did not confirm an association of the *AMPD1*:rs17602729 polymorphism with total survival rate or cardiac survival rate for HF. On the other hand, the rs17602729:c.34C>T transition in patients with cardiovascular diseases was associated by Feng et al. with higher left ventricular ejection fraction (LVEF), lower left ventricular end-diastolic dimension (LVEDD), and lower systolic blood pressure (SBP) [5]. It is also worth noting that our study from 2009 revealed that the *AMPD1*:rs17602729 polymorphism was associated with a decreased frequency of obesity in Polish patients with coronary artery disease (CAD) and with decreased diabetes prevalence in CAD patients with heart failure [1].

The *AMPD1*: rs34526199: c.860A>T transversion, identified in 2004 by Toyama et al. in German myopathic patients [6], results in the substitution of a highly conserved Lysine by Isoleucine at amino-acid position 287 (p.Lys287Ile) of AMP deaminase 1. The p.Lys287Ile variant, which affects the AMPD1 region responsible for myosin binding, decreases the enzyme activity by approximately half compared to that from the reference gene [7]. In 2011, Safranow et al. demonstrated that the prevalence of a variant *AMPD1*: rs34526199: c.860T allele in Polish CAD patients was significantly lower compared to control subjects; however, in contrast to the *AMPD1*:rs17602729 polymorphism [1], this genetic variant was associated in these patients with a higher prevalence of diabetes and a higher risk of abdominal obesity [7].

Therefore, taking into consideration the results obtained so far, we decided to analyze the possible associations of both *AMPD1*:rs17602729 and rs34526199 polymorphisms, and combinations of these, with clinical and biochemical phenotypes in diabetic individuals with CAD only.

## 2. Results

The *AMPD1*:rs17602729 and rs34526199 genotype distributions conformed to the expected Hardy–Weinberg equilibria both in the diabetic individuals with CAD (*p* = 0.261 and *p* = 0.526, respectively) and in the control group consisting of healthy full-term newborns (*p* = 0.330 and *p* = 0.531, respectively).

The frequency of the minor *AMPD1*:c.34T allele was 16.2% (34 out of 210 alleles) or 14.7% (28 out of 190 alleles) in either female or male controls, respectively. In female newborns there were 72 CC homozygotes (68.6%), 32 CT heterozygotes (30.5%), and 1 TT homozygote (0.9%), and in male controls, there were 69 CC homozygotes (72.6%), 24 CT heterozygotes (25.3%), and 2 TT homozygotes (2.1%). In the diabetic individuals with CAD, the frequency of the minor *AMPD1*:c.34T allele was 16.7% (18 out of 108 alleles) or 15.5% (44 out of 284 alleles) in female or male patients, respectively. In female patients there were 39 CC homozygotes (72.2%), 12 CT heterozygotes (22.2%), and 3 TT homozygotes (5.6%), and in diabetic male patients with CAD, there were 102 CC homozygotes (71.8%), 36 CT heterozygotes (25.3%), and 4 TT homozygotes (2.8%). No significant differences in the frequency distributions of *AMPD1*:rs17602729 alleles or genotypes have been found in regard to sex (females vs. males) both in the control group (*p* = 0.688 or *p* = 0.594) and in diabetic individuals with CAD (*p* = 0.776 or *p* = 0.613).

The frequency of the minor *AMPD1*:c.860T allele was 3.3% (7 out of 210 alleles) or 5.3% (10 out of 190 alleles) in either female or male controls, respectively. In female subjects, there were 98 AA homozygotes (93.3%) and 7 AT heterozygotes (6.7%), and in male controls, there were 85 AA homozygotes (89.5%) and 10 AT heterozygotes (10.5%). In diabetic individuals with CAD, the frequencies of the minor *AMPD1*:c.860T allele was 5.6% (6 out of 108 alleles) and 3.9% (11 out of 284 alleles) in female and male patients, respectively. In female patients, there were 48 AA homozygotes (88.9%) and 6 AT heterozygotes (11.1%), and in diabetic male patients with CAD, there were 131 AA homozygotes (92.3%) and 11 AT heterozygotes (7.7%). No significant differences in the frequency distributions of *AMPD1*: rs34526199 alleles or genotypes have been found in regard to sex (females vs. males) either in the control group (*p* = 0.447 or *p* = 0.339) or in diabetic individuals with CAD (*p* = 0.465 or *p* = 0.454).

There were no significant differences between both groups (controls vs. diabetic individuals with CAD in the frequency distributions of *AMPD1*:rs17602729 and rs34526199 alleles or genotypes (Table 1).

Apart from BMI and HDL cholesterol, there were no significant differences in the analyzed variables in the studied group of diabetic individuals with CAD in regard to the AMPD1:rs17602729 genotype (CC homozygotes vs. CT heterozygotes vs. TT homozygotes). Both BMI and the frequency of obesity (defined as BMI ≥ 30 kg/m^2^) in TT homozygotes were significantly lower compared to patients carrying at least one reference rs17602729:c.34C allele. In addition, serum concentrations of HDL cholesterol in patients homozygous for the c.34T allele were significantly higher compared to subjects with at least one c.34C allele. On the other hand, concentrations of total cholesterol and LDL cholesterol in patients with at least one variant c.34T allele (with CT or TT genotypes) were significantly higher compared to their values in reference *AMPD1*:rs17602729 (CC) homozygotes (Table 2).

Apart from age, no significant differences in all the analyzed variables were found with regard to AMPD1:rs34526199 genotype (AA homozygotes vs. AT heterozygotes. The AT heterozygotes were significantly older compared to patients homozygous in the reference c.860A allele (AA homozygotes) (Table 3).

There were no significant differences between diabetic individuals with CAD and control newborns in the frequency distributions for combinations of AMPD1:rs17602729/rs34526199 genotypes (Table 4). In addition, there were no significant differences between both groups in the frequency distributions of *AMPD1*:rs17602729/rs34526199 genotype combinations determining full enzyme activity (CC/AA), intermediate enzyme activity (CC/AT, CT/AA and CT/AT), or null enzyme activity (TT/AA) (Table 4).

Apart from BMI, the prevalence of obesity, and the serum concentrations of total cholesterol and HDL cholesterol, there were no significant differences in the analyzed variables in diabetic patients with CAD in regard to genetically predicted AMPD1 activity (full enzyme activity vs. intermediate enzyme activity vs. null enzyme activity).

The BMI and the frequency of obesity in patients with no AMPD1 activity were significantly lower compared to other patients (e.g., patients with full or intermediate activity of AMP deaminase 1). The concentrations of total cholesterol and LDL cholesterol in the latter patients were significantly lower compared to subjects with no AMPD1 activity. On the other hand, the concentrations of HDL cholesterol in serum of patients with lack of AMPD1 activity was significantly higher compared to subjects with full or intermediate activities of AMP deaminase 1 (Table 5).

## 3. Discussion

There were no statistically significant associations found for either *AMPD1*: rs17602729 and *AMPD1*: rs34526199 polymorphisms or AMP deaminase 1 (AMPD1) activity predicted with the risk of coronary artery disease in Polish individuals with diabetes. However, it is worth noting that the TT genotype of the rs17602729 polymorphism, which determines a lack of activity of this enzyme (see the Introduction), was associated with a lower BMI, a lower frequency of obesity, and a higher concentration of HDL cholesterol. In turn, reference homozygotes for this polymorphism (CC), as well as patients with normal AMPD1 activity, i.e., double reference *AMPD1* homozygotes (CC for rs17602729 and AA for rs34526199), were characterized by significantly lower total cholesterol and LDL cholesterol concentrations compared to the other subjects. For the *AMPD1*:rs34526199 polymorphism assessed separately, no association was found with any of the analyzed variables.

We had previously found no association of *AMPD1*: rs17602729 with coronary artery disease in another group of 201 Polish patients but the percentage of individuals with diabetes in this group was only 19% [1]. However, the *AMPD1*: rs34526199 (c.860A>T) polymorphism was associated with the risk of CAD in this group [7]. It is worth emphasizing that the control group in the previous study and in the current report consisted of 200 healthy full-term newborns from our region. It should be noted that healthy newborns have been widely used as control groups in similar association studies [8,9,10] as representative samples of the general population [11].

Furthermore, association studies using newborns as controls may have advantages due to the exclusion of confounding environmental influences such as diseases or lifestyle [12]. Furthermore, both the study and control groups consisted of subjects born in Polish Western Pomerania, descendants of people who came to this region after World War II from almost all regions of the former Second Polish Republic [13,14]. Therefore, contemporary inhabitants of Polish Western Pomerania are considered a representative sample for the Polish population in genetic epidemiological studies [15,16]. The random selection of newborns born in one of the hospitals in Szczecin for the control group additionally minimized the risk of population bias and stratification [16]. It is worth noting here that the frequency of c.34T *AMPD1* allele in our newborn controls (15.5%) was very close to its prevalence in the combined control group consisted of adult healthy Poles (15.7%) [17,18,19,20]. The frequency of c.34T variant in Polish subjects is also similar to its prevalence in Czechs, another Slavic population (16.4%) [21], as well as to non-Slavic Europeans in the 1000 Genomes (1KG) project (8.9–14.3%) (https://www.ensembl.org/Homo_sapiens/Variation/Population?db=core;r=1:114692936-114693936;v=rs17602729;vdb=variation;vf=722185, accessed on 4 May 2025). On the other hand, the low frequency of *AMPD1*: c.34T variant in non-European 1KG populations (ranging from 5.8% in Americans to 0.1% in East Asians) seems to suggest that the protein-truncating *AMPD1*:rs17602729 mutation (p.Gln12Ter) may modify the susceptibility to specific cardiovascular or metabolic intermediate phenotypes, but only in Europeans.

While for the rs17602729 polymorphism of the *AMPD1* gene the frequency of the variant allele c.34T in the previously analyzed group of CAD patients was similar to its frequency in the current study (16.9% and 15.8%, respectively), it is difficult to explain the differences between these groups for the *AMPD1*:rs34526199 (c.860A>T) polymorphism where the frequency of the c.860T allele was 1.7% and 4.3%, respectively.

We assume that among the potential confounding factors, the differences in the phenotypic characteristics of both groups of CAD patients should be taken into account above all in age, sex ratio, obesity frequency, or diabetes frequency [1,7]. The frequency of the c.860T allele found by us in diabetic patients with CAD (4.3%) and in the neonatal control group (4.2%) is very close to the average frequency of this variant in other European countries analyzed in the 1000 Genomes project (3.7%) (https://www.ensembl.org/Homo_sapiens/Variation/Population?db=core;r=1:114679116−114680116;v=rs34526199;vdb=variation;vf=779537, accessed on 4 May 2025).

The results of the present work have confirmed our previous findings regarding the association of the c.34C>T *AMPD1* polymorphism with BMI and obesity [1]. However, a novel finding currently revealed here is the association of genetically predicted (and mainly determined by the rs17602729 polymorphism) AMP deaminase 1 activity with serum concentrations of total cholesterol, LDL cholesterol, and HDL cholesterol. We found that the lack of AMPD1 activity (with TT homozygotes of the rs17602729 polymorphism) was associated with higher HDL cholesterol levels compared to the other patients, and on the other hand, the normal activity of this enzyme (determined by simultaneous homozygosity for the reference alleles of the c.34C>T polymorphism and the c.860A>T polymorphism) was associated with lower levels of total cholesterol and LDL cholesterol compared to patients with reduced AMPD1 activity or a even complete lack of enzyme activity. This association does not seem to be related to obesity, the degree of diabetes control or statin use, because in terms of these three variables, we did not find any significant differences between patients with different *AMPD1*:rs17602729 genotypes (i.e., with different AMP deaminase 1 activities).

The revealed associations are particularly intriguing, because in our previous analysis in patients with CAD we did not detect such link [1], perhaps because of the much lower number of diabetic CAD patients. In addition, the lack of association between *AMPD1*: rs17602729 polymorphism and lipid parameters in young, non-obese Polish women without metabolic disorders was also reported in 2020 by Leońska-Duniec et al. [17]. However, it is worth noting that our current study concerned only diabetic patients with CAD, whilst in our previous analysis carried out in patients with CAD, the percentage of diabetic subjects was only 19% [1], and Leoniec-Duńska et al. solely analyzed non-diabetic women [17]. Therefore, taking the above into account, we cannot exclude that the association of *AMPD1*:rs rs17602729 with cholesterol concentrations may be limited only to diabetic patients.

Smolenski et al. emphasized that the reduction in AMP deaminase 1 activity caused by the premature introduction of the termination codon as a result of the c.34C>T variant of the *AMPD1* gene leads to the accumulation of AMP and, consequently, the activation of 5′ AMP-activated protein kinase (AMPK) [22]. Activated AMPK subsequently acts on a broad range of signaling pathways involved in metabolic regulation, e.g., cholesterol synthesis, fatty acid transport and oxidation, insulin secretion, glucose uptake, lipogenesis, or lipolysis [23,24]. AMPK not only acts directly on various enzymes involved in these metabolic pathways but also at the transcriptional level of many genes acting in glucose and lipid metabolism [25]. In this way, AMPK controls cellular energy homeostasis, switching off ATP consumption (e.g., protein synthesis, cholesterol synthesis) and initiating ATP- and energy-producing processes (e.g., lipid oxidation, glucose uptake) in times of nutrient shortage [26].

However, the fact that our results appear to indicate that genetically predicted reduced AMPD1 activity in CAD patients with diabetes is rather associated with increased serum concentrations of total cholesterol, LDL cholesterol, and HDL cholesterol seems to contradict the above explanation and suggests that the molecular mechanisms of this metabolic pathway are more complex.

Firstly, there are three isoenzymes of human AMP deaminase exhibiting different physical, catalytic, and regulatory properties [27]. Besides AMP deaminase 1, encoded by the *AMPD1* gene at chromosome 1p13.2, two other AMPD isoenzymes, i.e., the liver-specific AMP deaminase 2, encoded by the A*MPD2* gene at chromosome 1p13.3, and the erythrocyte-specific AMP deaminase 3, encoded by the *AMPD3* gene at chromosome 11p15.4., have been identified [27]. It is also worth noting that, in 2017, Christie et al. discovered an association of lipid concentrations with an *AMPD3* polymorphism but not with *AMPD1* variants [28]. These authors showed that the *AMPD3*:rs2923084 polymorphism was associated with HDL cholesterol concentrations in 5-year-old children [24]. According to Żydowo, the enzyme encoded by the *AMPD3* gene interacts with lipids in the cytosol regulation process [29].

Secondly, AMPK is a heterotrimeric enzyme, comprising one catalytic α-subunit (two isoforms, α1 and α2, encoded by *PRKAA1* and *PRKAA2* genes, respectively), one regulatory β-subunit (two isoforms, β1 and β2, encoded by *PRKAB1* and *PRKAB2* genes, respectively) and one regulatory γ-subunit (three isoforms,, γ1, γ2, and γ3 encoded by *PRKAG1*, *PRKAG2*, and *PRKAG32* genes, respectively). The isoforms of all three subunits are encoded by separate genes [30] and plausible evidence suggests that the beneficial effects of AMPK activation on serum lipid profiles in humans can be influenced by genetic variation in AMPK subunits [30,31,32]. Spencer-Jones et al. revealed the association of haplotypes based on five tagging SNPs in the *PRKAA2* gene with total, LDL, and HDL cholesterol in 2777 normal female individuals of European descent [30]. In addition, Weyrich et al. found that the minor alleles of rs692243 and rs6436094 polymorphisms in *PRKAG3* gene were associated with higher serum LDL cholesterol and apolipoprotein B-100 levels in 1061 non-diabetic Germans [31]. Finally, Randrianarisoa et al. identified six largely nonoverlapping SNP sets across four AMPK genes (*PRKAA1, PRKAA2, PRKAG2*, and *PRKAG3*) associated with adiposity, insulin sensitivity, insulin secretion, and blood glucose, as well as with total, LDL, and HDL cholesterol, in a cohort of 2789 nondiabetic participants from the Tübingen Family study of type 2 diabetes [32].

A major limitation of our study is its relatively low statistical power resulting from a small sample size. Using an open-source software Open Epi (www.openepi.com), we have computed the minimum sample size for 80% statistical power and a 5% type I error rate (α). We have assumed a ratio of control newborns to CAD diabetics equal to 1.02 (200/196) and a frequency of subjects homozygous for the T allele of *AMPD1*:rs17602729 equal to 3.5% in the studied group and 1.5% in the control group. Under these assumptions, the estimated minimum sample size in a recessive mode of inheritance of the T allele was 965 or 946 for newborn controls or CAD diabetics, respectively.

## 4. Materials and Methods

### 4.1. Patients and Control Subjects

The study group consisted of 196 adult patients (54 women and 142 men, aged 40–70 years old), recruited in the outpatient clinic of the University Clinical Hospital No. 2 in Szczecin, Poland. The criteria for inclusion into the study group were individuals with type 2 diabetes mellitus (T2DM) and angiographically documented coronary artery disease (CAD). Clinical data from patients’ records included the following: age; sex; body mass; body height; body mass index (BMI), calculated as body mass (kg)/(height (m)^2^); waist and hip circumferences; waist–hip ratio (WHR); previous myocardial infarction; and presence of arterial hypertension and statin use. Laboratory data from patients’ records included the following: fasting plasma glucose levels, levels of glycated hemoglobin (HbA1c), serum concentrations of creatinine and estimated Glomerular Filtration Rate (eGFR) calculated using the CKD-EPI equation, serum concentrations of triglycerides (TG), total cholesterol (TC), LDL cholesterol (LDL-C), and HDL cholesterol (HDL-C). From all patients, peripheral blood samples (5 mL) were drawn and stored at −20 °C until DNA isolation. The population-based control group for genetic case–control analysis consisted of 200 newborns (104 girls and 96 boys) described previously elsewhere [1]. The control subjects were randomly chosen from the Newborn DNA Repository at the Department of Clinical and Molecular Biochemistry, Pomeranian Medical University in Szczecin. All DNA samples in our Repository were collected in breastfed and medication-free neonates. Twins and infants of mothers with diabetes, preeclampsia, hypertension of any cause, history of illicit substance use, or antenatal steroid therapy were excluded. Other exclusion criteria were congenital infection, intrauterine growth restriction (i.e., below the 10th percentile birth mass, length, or head circumference), chromosomal aberrations, or congenital malformations. Both patients and control newborns were Poles of European descent. The study was conducted in accordance with the latest Declaration of Helsinki (2013) and was approved by the bioethics committee at the Pomeranian Medical University in Szczecin, Poland. All patients and parents of all newborns gave informed, written consent for participation in the study.

### 4.2. AMPD1 Genotyping

Genomic DNA was isolated either from peripheral blood leukocytes (patients) or from umbilical cord blood leukocytes (newborn infants) using a commercially available DNA isolation kit (QIAamp Blood DNA Mini Kit, QIAGEN, Hilden, Germany). Both *AMPD1* SNPs (rs17602729 and rs34526199) were identified by SNaPshot minisequencing reactions. PCR and SNaPshot primers were designed using *PrimerSelect* software v.11 (DNASTAR, Inc. Madison, WI, USA). For *AMPD1*: rs17602729 genotyping the following oligonucleotides were carried out: 5′-GGAAGGCTGAGCTGAAATAACA-3′ as the PCR forward primer, 5′-TGA CAAATGGCAGCAAAAGTAA-3′ as the PCR reverse primer, and 5′-GACTGACTGACT GACTTACTTCATACAGCTGAAGAGAAA-3′ as the SnaPshot primer. For A*MPD1*: rs34526199 genotyping following oligonucleotides were carried out: 5′-TCTTGAATGCCTG AAACTT-3′ as the PCR forward primer, 5′-ATGGGGAAAACGATAGAA-3′ as the PCR reverse primer, and 5′-GACTGACTGACTGACTGACTGGACGAGTTAAAGGAGCTGA-3′ as the SNaPshot primer. PCR amplification was carried out in a total volume of 20 μL containing 2 μL DNA (80 ng); 10 μL 2× PCR Master Mix (Thermo Scientific Fermentas, Waltham, MA, USA); 0.2 μL PCR primer forward [20 pmol/μL]; 0.2 μL PCR primer reverse [20 pmol/μL]; and 7.6 μL H_2_O. Thermal cycling was carried out in a Mastercycler Pro S (Eppendorf), with the following conditions: 94 °C pre-incubation step for 5 min (min); 38 cycles of 94 °C denaturation for 20 s, annealing at (62.5 °C or 52 °C, for rs17602729 or rs34526199, respectively) for 40 s and extension at 72 °C for 40 s; followed by 10 min for the final extension at 72 °C and then 4 °C until removed from the thermocycler. PCR products and negative controls were checked by agarose gel electrophoresis. PCR products were purified to remove excess primers and unincorporated dNTPs by adding 1 μL [20 U/μL] of Exonuclease I and 2 μL [1 U/μL] of FastAP™ Thermosensitive Alkaline Phosphatase (Thermo Scientific Fermentas) to each 10 μL PCR product. Reactions were incubated at 37 °C for 15 min followed by 80 °C for 15 min for enzyme deactivation. The minisequencing reaction was performed using SNaPshot™ Multiplex Kit (Applied Biosystems, Waltham, MA, USA). The minisequencing reaction was carried out in a Mastercycler Pro S (Eppendorf, Hamburg, Germany) using 2.5 μL of the SNaPshot™ Multiplex Ready Reaction Mix (Applied Biosystems), 1 μL purified PCR product, 1 μL H_2_O, and 0.5 μL of SNaPshot primer [0.2 pmol/μL] for a final volume of 5 μL. The PCR conditions were 25 cycles of denaturation at 96 °C for 10 s, annealing at 50 °C for 5 s, and extension at 60 °C for 30 s. To remove the unincorporated ddNTPs, the final product was treated with 0.5 μL [1 U/μL] of Fast AP Thermosensitive Alkaline Phoshatase (Thermo Scientific Fermentas) at 37 °C for 60 min followed by 75 °C for 15 min for enzyme inactivation. Minisequencing products (0.5 μL) were mixed with 10 μL of Hi-Di™ Formamide and 0.15 μL of GeneScan-120 LIZ (both Applied Biosystems). Samples were denatured at 95 °C for 5 min and quickly cooled on ice for 5 min. Electrophoresis was run on an ABI PRISM^®^ 3130 Genetic Analyzer (Applied Biosystems). Resulting data were analyzed using *GeneMapper^®^ ID* software v4.1 (Applied Biosystems).

### 4.3. Statistical Analyses

As the distributions of quantitative variables, tested using Shapiro–Wilk tests, were significantly different from normal distributions, these values were compared between groups using non-parametric Kruskal–Wallis tests or Mann–Whitney tests, if necessary. The concordance of the distribution of *AMPD1* genotypes with Hardy–Weinberg equilibrium (HWE) and qualitative variables, including the frequencies of genotypes and alleles, were assessed using chi-square tests. In addition, all variables were analyzed using Mann–Whitney tests or chi-square tests (quantitative variables or qualitative variables, respectively) using dominant or recessive modes of inheritance for the risk (minor) alleles: *AMPD1*: rs17602729: c.34T and rs34526199: c.860T alleles) or for combinations involving genotype-predicted AMPD1 activity (full AMPD1 activity vs. others or no AMPD1 activity vs. others). A *p*-value < 0.05 was considered statistically significant. All data were analyzed using a data analysis software system (Dell Statistica, version 13. Dell Inc., Round Rock, TX, USA, 2016, software.dell.com).

## 5. Conclusions

Our results suggest that genetically predicted AMP deaminase 1 activity is associated with variation in body mass and lipid metabolism in Polish individuals with diabetes and coronary artery disease.

## Figures and Tables

**Table 1:** Frequency distributions of *AMPD1*: rs17602729 (c.34C>T) and rs34526199 (c.860A>T) alleles or genotypes in diabetic individuals with CAD and in newborn controls.

Polymorphism (Chromosomal Location ^a^)	Allele ^b^	Distribution of Alleles, n _A_ (%)		*p*	Distribution of Genotypes ^b^, n _G_ (%)						*p*	*p*_R_	*p*_D_
	(1/2)	Diabetic Individuals with CAD	Newborn Controls		Diabetic Individuals with CAD			Newborn Controls					
		1/2	1/2		[1;1]	[1(;)2]	[2;2]	[1;1]	[1(;)2]	[2;2]			
rs17602729 (1:114693436)	C/T	330/62 (84.2/15.8)	338/62 (84.5/15.5)	0.903	141 (71.9)	48 (24.5)	7 (3.6)	141 (70.5)	56 (28.0)	3 (1.5)	0.338	0.189	0.752
rs34526199 (1:114679616)	A/T	375/17 (95.7/4.3)	383/17 (95.8/4.2)	0.952	179 (91.3)	17 (8.7)	0 (0.0)	183 (91.5)	17 (8.5)	0 (0.0)	0.951	-	-

^a^ Single nucleotide polymorphism location was indexed to NCBI build 38 (GRCh38.p13). ^b^ Alleles 1 and 2 were defined as the major and minor (rarer) alleles, respectively. n _A_—number of alleles; n _G_—number of genotypes; *p*—significance values for chi^2^ 2 × 2 table (alleles) or for chi^2^ 2 × 3 table (genotypes). *p*_R_ or *p*_D_—significance values in recessive or dominant modes of inheritance for the minor allele (allele 2), respectively.

**Table 2:** Characteristics of diabetic patients with CAD in regard to *AMPD1*: rs17602729 (c.34C>T) polymorphism.

Variable	All	*AMPD1*: rs17602729 (c.34C>T) Genotype			*p*
	(n = 196)	CC (n = 141)	CT (n = 48)	TT (n = 7)	
Female, n (%)	54 (27.0)	39 (27.6)	12 (25.0)	3 (42.8)	0.613
Myocardial infarction, n (%)	98 (50.0)	72 (51.1)	21 (43.7)	5 (71.4)	0.351
Arterial hypertension, n (%)	184 (93.9)	132 (93.6)	45 (93.7)	7 (100.0)	0.789
Heart failure, n (%)	27 (13.8)	21 (14.9)	5 (10.4)	1 (14.3)	0.739
Statin use, n (%)	170 (86.7)	119 (84.4)	44 (91.7)	7 (100.0)	0.289
Metformin use	139 (70.9)	102 (72.3)	33 (68.7)	4 (57.1)	0.640
Sulphonylureas use	45 (22.9)	28 (19.8)	15 (31.2)	2 (28.6)	0.252
Insulin use	79 (40.3)	60 (42.6)	16 (33.3)	3 (42.9)	0.526
Age (years)	66 (44:84)	66 (44:82)	64 (50:80)	69 (55:84)	0.381
Body height (cm)	168 (135:183)	168 (135:182)	167 (147:183)	170 (158:182)	0.806
Body mass (kg)	86 (53:139)	86 (53:121)	89 (65:139)	73 (62:110)	0.115
BMI (kg/m^2^)	30.9 (20.4:45.7)	30.7 (20.4:45.7)	31.5 (22.8:41.6)	26.3 (24.1:35.1) ^A^	0.008
BMI ≥ 30 kg/m^2^, n (%)	114 (58.2)	81 (57.4)	32 (66.7)	1 (14.3) ^B^	0.031
Waist (cm)	106 (71:138)	105 (72:128)	107 (71:138)	92 (83:115)	0.278
Hip (cm)	104 (90:134)	104 (90:134)	106 (92:125)	101 (94:113)	0.077
WHR	0.99 (0.77:1.17)	1.00 (0.77:1.14)	0.97 (0.77:1.16)	0.94 (0.78:1.17)	0.292
Fasting glucose (mg/dL)	132 (67:356)	134 (67:356)	127 (71:308)	126 (103:353)	0.866
HbA_1C_ (%)	6.6 (4.7:11.8)	6.8 (4.8:11.7)	6.4 (4.7:11.8)	6.9 (5.5:10.8)	0.579
Creatinine (mg/dL)	0.91 (0.61:6.67)	0.91 (0.61:5.34)	0.89 (0.67:6.67)	0.86 (0.76:1.37)	0.767
e-GFR_CKD EPI_ (ml/min/1.73 m^2^)	80.9 (7.7:111.5)	79.5 (10.4:111.5)	82.2 (7.7:102.5)	81.8 (40.0:103.1)	0.509
TC (mg/dL)	164 (101:358)	154 (101:358) ^C^	184 (101:309)	192 (153:272)	0.070
HDL-C (mg/dL)	43 (20:69)	41 (26:69)	41 (20:61)	57 (47:63) ^D^	0.031
LDL-C (mg/dL)	98 (34:298)	89 (41:298) ^E^	117 (34:218)	126 (68:169)	0.110
TG (mg/dL)	151 (41:821)	138 (44:821)	162 (65:485)	123 (41:318)	0.296

Quantitative data are presented as median (minimum/maximum). BMI—body mass index; CAD—coronary artery disease; e-GFR_CKD EPI_—estimated glomerular filtration rate according to CKD-EPI (Chronic Kidney Disease Epidemiological Collaboration) equation; HbA_1C_—glycated hemoglobin; HDL—HDL cholesterol; LDL—LDL cholesterol; NYHA—New York Heart Association; TC—total cholesterol; TG—triacylglycerols; WHR—waist-to-hip ratio. ^A^ *p* = 0.009, comparing TT homozygotes to patients with at least one C allele (CC or CT); ^B^ *p* = 0.017, comparing TT homozygotes to patients with at least one C allele (CC or CT); ^C^ *p* = 0.022, comparing CC homozygotes to patients with at least one T allele (CT or TT); ^D^ *p* = 0.01, comparing TT homozygotes to patients with at least one C allele (CC or CT); ^E^ *p* = 0.037, comparing CC homozygotes to patients with at least one T allele (CT or TT).

**Table 3:** Characteristics of diabetic patients with CAD in regard to *AMPD1*: rs34526199 (c.860A>T) polymorphism.

Variable	*AMPD1*: rs34526199 (c.860A>T) Genotype		*p*
	AA (n = 179)	AT (n = 17)	
Female, n (%)	48 (26.8)	6 (35.3)	0.570
Myocardial infarction, n (%)	91 (50.8)	7 (41.2)	0.613
Arterial hypertension, n (%)	168 (93.8)	16 (94.1)	1.000
Heart failure, n (%)	27 (15.1)	0 (0.0)	0.175
Statin use, n (%)	153 (85.5)	17 (100.0)	0.136
Metformin use	126 (70.4)	13 (76.5)	0.598
Sulphonylureas use	41 (22.9)	4 (23.5)	0.953
Insulin use	72 (40.2)	7 (41.2)	0.940
Age (years)	66 (44:84)	69 (56:81)	0.031
Body height (cm)	169 (135:183)	165 (143:178)	0.072
Body mass (kg)	86 (53:139)	86 (55:121)	0.592
BMI (kg/m^2^)	30.8 (20.4:41.6)	32.0 (22.9:45.7)	0.375
BMI ≥ 30 kg/m^2^, n (%)	104 (58.1)	10 (58.9)	0.556
Waist (cm)	105 (71:138)	107 (78:128)	0.683
Hip (cm)	104 (90:130)	104 (96:134)	0.991
WHR	0.99 (0.77:1.17)	0.99 (0.82:1.11)	0.852
Fasting glucose (mg/dL)	133 (67:356)	128 (81:237)	0.291
HbA_1C_ (%)	6.7 (4.7:11.8)	6.5 (5.4:8.9)	0.626
Creatinine (mg/dL)	0.90 (0.65:6.67)	0.97 (0.61:1.90)	0.834
e-GFR_CKD EPI_ (ml/min/1.73 m^2^)	81.1 (7.7:111.5)	80.7 (33.4:92.0)	0.521
TC (mg/dL)	164 (101:358)	168 (140:337)	0.610
HDL-C (mg/dL)	42 (20:69)	55 (41:56)	0.183
LDL-C (mg/dL)	96 (34:298)	98 (62:226)	0.682
TG (mg/dL)	146 (41:821)	170 (96:249)	0.383

Quantitative data are presented as median (minimum/maximum). BMI—body mass index; CAD—coronary artery disease; e-GFR_CKD EPI_—estimated glomerular filtration rate according to CKD-EPI (Chronic Kidney Disease Epidemiological Collaboration) equation; HbA_1C_—glycated hemoglobin; HDL—HDL cholesterol; LDL—LDL cholesterol; NYHA—New York Heart Association; TC—total cholesterol; TG—triacylglycerols; WHR—waist-to-hip ratio.

**Table 4:** Frequency distribution of *AMPD1*: rs17602729 (c.34C>T)/rs34526199 (c.860A>T) combined genotypes in diabetic patients with CAD and in newborn controls.

	Distribution of *AMPD1*: rs17602729/rs34526199 Combined Genotypes, n (%)					*p* *	*p* #
	CC/AA	CC/AT	CT/AA	CT/AT	TT/AA		
Diabetic Individuals with CAD	126 (64.3)	15 (7.6)	46 (23.5)	2 (1.0)	7 (3.6)	0.604	0.339
Newborn Controls	125 (62.5)	16 (8.0)	55 (27.5)	1 (0.5)	3 (1.5)		

* significance value for chi^2^ 2 × 5 table; # significance value for chi^2^ 2 × 3 table (full AMPD1 activity: CC/AA vs. intermediate AMPD1 activity: CC/AT+CT/AA+CT/AT vs. no AMPD1 activity: TT/AA).

**Table 5:** Characteristics of diabetic patients with CAD in regard to genotype-predicted AMP deaminase 1 (AMPD1) activity.

Variable	Genotype-Predicted AMPD1 Activity			*p*
	Full (n = 126)	Intermediate (n = 63)	No Activity (n = 7)	
Female, n (%)	33 (26.2)	18 (28.6)	3 (42.8)	0.616
Myocardial infarction, n (%)	65 (51.6)	28 (44.4)	5 (71.4)	0.335
Arterial hypertension, n (%)	118 (93.6)	59 (93.6)	7 (100.0)	0.789
Heart failure, n (%)	21 (16.7)	5 (7.9)	1 (14.3)	0.260
Statin use, n (%)	104 (82.5)	59 (93.6)	7 (100.0)	0.061
Metformin use	91 (72.2)	44 (69.8)	4 (57.1)	0.676
Sulphonylureas use	30 (23.8)	13 (20.6)	2 (28.6)	0.832
Insulin use	53 (42.1)	23 (36.5)	3 (42.9)	0.756
Age (years)	66 (44:82)	66 (50:81)	69 (55:84)	0.620
Body height (cm)	169 (135:182)	167 (143:183)	170 (158:182)	0.332
Body mass (kg)	86 (53:119)	88 (55:139)	73 (62:110)	0.177
BMI (kg/m^2^)	30.7 (20.3:40.3)	31.7 (22.8:45.7)	26.3 (24.1:35.1) ^A^	0.004
BMI ≥ 30 kg/m^2^, n (%)	72 (57.2)	41 (65.1)	1 (14.3) ^B^	0.033
Waist (cm)	105 (72:125)	107 (71:138)	92 (83:115)	0.255
Hip (cm)	104 (90:130)	106 (92:134)	101 (94:113)	0.087
WHR	1.00 (0.77:1.14)	0.98 (0.77:1.16)	0.94 (0.78:1.17)	0.326
Fasting glucose (mg/dL)	137 (67:356)	127 (71:308)	126 (103:353)	0.319
HbA_1C_ (%)	6.8 (4.8:11.7)	6.4 (4.7:11.8)	6.9 (5.5:10.8)	0.376
Creatinine (mg/dL)	0.91 (0.65:5.34)	0.91 (0.61:6.67)	0.86 (0.76:1.37)	0.659
e-GFR_CKD EPI_ (ml/min/1.73 m^2^)	79.4 (10.4:111.5)	81.6 (7.7:102.5)	81.8 (40.0:103.1)	0.799
TC (mg/dL)	152 (101:358) ^C^	184 (101:337)	192 (153:272)	0.035
HDL-C (mg/dL)	41 (26:69)	42 (20:61)	57 (47:63) ^D^	0.022
LDL-C (mg/dL)	87 (41:298) ^E^	117 (34:226)	126 (68:169)	0.058
TG (mg/dL)	132 (44:821)	167 (65:485)	123 (41:318)	0.116

Full AMPD1 activity was predicted for patients with reference homozygous genotypes for *AMPD1*:rs17602729 (CC) and for rs34526199 (AA). No AMPD1 activity was predicted for patients with *AMPD1*:rs17602729 TT genotype and *AMPD1*:rs34526199 AA or AT genotypes, respectively. The group of patients with intermediate AMPD1 activity consisted of subjects with other *AMPD1* genotypes for both polymorphisms. Quantitative data are presented as median (minimum/maximum). BMI—body mass index; CAD—coronary artery disease; e-GFR_CKD EPI_—estimated glomerular filtration rate according to CKD-EPI (Chronic Kidney Disease Epidemiological Collaboration) equation; HbA_1C_—glycated hemoglobin; HDL—HDL cholesterol; LDL—LDL cholesterol; NYHA—New York Heart Association; TC—total cholesterol; TG—triacylglycerols; WHR—waist-to-hip ratio. ^A^ *p* = 0.009, comparing patients with lack of AMPD1 activity to other patients; ^B^ *p* = 0.017, comparing patients with full AMPD1 activity to other patients; ^C^ *p* = 0.01, comparing patients with full AMPD1 activity to other patients; ^D^ *p* = 0.01, comparing patients with lack of AMPD1 activity to other patients; ^E^ *p* = 0.02, comparing patients with full AMPD1 activity to other patients.

## Data Availability

The data are available from the corresponding author upon reasonable request.

## Abbreviations

The following abbreviations are used in this manuscript:

*AMPD1*	gene encoding adenosine monophosphate deaminase 1
*AMPD2*	gene encoding adenosine monophosphate deaminase 2
AMP	adenosine monophosphate
AMPDs	adenosine monophosphate deaminases
AMPK	5′ AMP-activated protein kinase
BMI	body mass index
CAD	coronary artery disease
CKD-EPI	Chronic Kidney Disease Epidemiological Collaboration
e-GFR	estimated glomerular filtration rate
HbA_1C_	glycated hemoglobin
HDL	HDL cholesterol
LDL	LDL cholesterol
NYHA	New York Heart Association
TC	total cholesterol
TG	triacylglycerols
WHR	waist-to-hip ratio

## References

[1] Safranow K., Czyzycka E., Binczak-Kuleta A., Rzeuski R., Skowronek J., Wojtarowicz A., Jakubowska K., Olszewska M., Loniewska B., Kaliszczak R. (2009). Association of C34T AMPD1 gene polymorphism with features of metabolic syndrome in patients with coronary artery disease or heart failure. Scand. J. Clin. Lab. Investig..

[2] Morisaki T., Sabina R.L., Holmes E.W. (1990). Adenylate deaminase. A multigene family in humans and rats. J. Biol. Chem..

[3] Morisaki T., Gross M., Morisaki H., Pongratz D., Zöllner N., Holmes E.W. (1992). Molecular basis of AMP deaminase deficiency in skeletal muscle. Proc. Natl. Acad. Sci. USA.

[4] Loh E., Rebbeck T.R., Mahoney P.D., DeNofrio D., Swain J.L., Holmes E.W. (1999). Common variant in AMPD1 gene predicts improved clinical outcome in patients with heart failure. Circulation.

[5] Feng A.F., Liu Z.H., Zhou S.L., Zhao S.Y., Zhu Y.X., Wang H.X. (2017). Effects of AMPD1 gene C34T polymorphism on cardiac index, blood pressure and prognosis in patients with cardiovascular diseases: A meta-analysis. BMC Cardiovasc. Disord..

[6] Toyama K., Morisaki H., Kitamura Y., Gross M., Tamura T., Nakahori Y., Vance J.M., Speer M., Kamatani N., Morisaki T. (2004). Haplotype analysis of human AMPD1 gene: Origin of common mutant allele. J. Med. Genet..

[7] Safranow K., Suchy J., Jakubowska K., Olszewska M., Bińczak-Kuleta A., Kurzawski G., Rzeuski R., Czyżycka E., Łoniewska B., Kornacewicz-Jach Z. (2011). AMPD1 gene mutations are associated with obesity and diabetes in Polish patients with cardiovascular diseases. J. Appl. Genet..

[8] Arking D.E., Krebsova A., Macek M., Macek M., Arking A., Mian I.S., Fried L., Hamosh A., Dey S., McIntosh I. (2002). Association of human aging with a functional variant of klotho. Proc. Natl. Acad. Sci. USA.

[9] Cybulski C., Górski B., Huzarski T., Byrski T., Gronwald J., Debniak T., Wokolorczyk D., Jakubowska A., Kowalska E., Oszurek O. (2006). CHEK2-positive breast cancers in young Polish women. Clin. Cancer Res..

[10] Freiberger T., Vyskocilová M., Kolárová L., Kuklínek P., Krystůfková O., Lahodná M., Hanzlíková J., Litzman J. (2002). Exon 1 polymorphism of the B2BKR gene does not influence the clinical status of patients with hereditary angioedema. Hum. Immunol..

[11] Larsen T.B., Lassen J.F., Brandslund I., Byriel L., Petersen G.B., Nørgaard-Pedersen B. (1998). The Arg506Gln mutation (FV Leiden) among a cohort of 4188 unselected Danish newborns. Thromb. Res..

[12] Han T., Wang X., Cui Y., Ye H., Tong X., Piao M. (2007). Relationship between angiotensin-converting enzyme gene insertion or deletion polymorphism and insulin sensitivity in healthy newborns. Pediatrics.

[13] Ploski R., Wozniak M., Pawlowski R., Monies D.M., Branicki W., Kupiec T., Kloosterman A., Dobosz T., Bosch E., Nowak M. (2002). Homogeneity and distinctiveness of Polish paternal lineages revealed by Y chromosome microsatellite haplotype analysis. Hum. Genet..

[14] Kayser M., Lao O., Anslinger K., Augustin C., Bargel G., Edelmann J., Elias S., Heinrich M., Henke J., Henke L. (2005). Significant genetic differentiation between Poland and Germany follows present-day political borders, as revealed by Y-chromosome analysis. Hum. Genet..

[15] Debniak T., Scott R.J., Huzarski T., Byrski T., Rozmiarek A., Debniak B., Załuga E., Maleszka R., Kładny J., Górski B. (2005). CDKN2A common variants and their association with melanoma risk: A population-based study. Cancer Res..

[16] Adler G., Łoniewska B., Parczewski M., Kordek A., Ciechanowicz A. (2009). Frequency of common CYP3A5 gene variants in healthy Polish newborn infants. Pharmacol. Rep..

[17] Leońska-Duniec A., Maculewicz E., Humińska-Lisowska K., Maciejewska-Skrendo A., Leźnicka K., Cięszczyk P., Sawczuk M., Trybek G., Wilk M., Lepionka W. (2020). *AMPD1* C34T Polymorphism (rs17602729) Is Not Associated with Post-Exercise Changes of Body Weight, Body Composition, and Biochemical Parameters in Caucasian Females. Genes.

[18] Ciȩszczyk P., Eider J., Ostanek M., Leońska-Duniec A., Ficek K., Kotarska K., Girdauskas G. (2011). Is the C34T polymorphism of the AMPD1 gene associated with athlete performance in rowing?. Int. J. Sports Med..

[19] Cieszczyk P., Ostanek M., Leońska-Duniec A., Sawczuk M., Maciejewska A., Eider J., Ficek K., Sygit K., Kotarska K. (2012). Distribution of the AMPD1 C34T polymorphism in Polish power-oriented athletes. J. Sports Sci..

[20] Gronek P., Gronek J., Lulińska-Kuklik E., Spieszny M., Niewczas M., Kaczmarczyk M., Petr M., Fischerova P., Ahmetov I.I., Żmijewski P. (2018). Polygenic Study of Endurance-Associated Genetic Markers *NOS3* (Glu298Asp), *BDKRB2* (-9/+9), *UCP2* (Ala55Val), *AMPD1* (Gln45Ter) and *ACE* (I/D) in Polish Male Half Marathoners. J. Hum. Kinet..

[21] Petr M., Thiel D., Kateřina K., Brož P., Malý T., Zahálka F., Vostatková P., Wilk M., Chycki J., Stastny P. (2022). Speed and power-related gene polymorphisms associated with playing position in elite soccer players. Biol. Sport.

[22] Smolenski R.T., Rybakowska I., Turyn J., Romaszko P., Zabielska M., Taegtmeyer A., Słomińska E.M., Kaletha K.K., Barton P.J. (2014). AMP deaminase 1 gene polymorphism and heart disease-a genetic association that highlights new treatment. Cardiovasc. Drugs Ther..

[23] Winder W.W., Hardie D.G. (1999). AMP-activated protein kinase, a metabolic master switch: Possible roles in type 2 diabetes. Am. J. Physiol..

[24] Steinberg G.R., Macaulay S.L., Febbraio M.A., Kemp B.E. (2006). AMP-activated protein kinase--the fat controller of the energy railroad. Can. J. Physiol. Pharmacol..

[25] Leff T. (2003). AMP-activated protein kinase regulates gene expression by direct phosphorylation of nuclear proteins. Biochem. Soc. Trans..

[26] Long Y.C., Zierath J.R. (2006). AMP-activated protein kinase signaling in metabolic regulation. J. Clin. Investig..

[27] Szydłowska M. (2005). Rodzina genów deaminazy AMP [Family of AMP-deaminase genes]. Postepy. Hig. Med. Dosw..

[28] Christie S., Robiou-du-Pont S., Anand S.S., Morrison K.M., McDonald S.D., Paré G., Atkinson S.A., Teo K.K., Meyre D. (2017). Genetic contribution to lipid levels in early life based on 158 loci validated in adults: The FAMILY study. Sci. Rep..

[29] Zydowo M.M. (1993). Regulatory effects of the lipid-cytosolic enzyme interaction: AMP deaminase. Acta Biochim. Pol..

[30] Spencer-Jones N.J., Ge D., Snieder H., Perks U., Swaminathan R., Spector T.D., Carter N.D., O’Dell S.D. (2006). AMP-kinase alpha2 subunit gene PRKAA2 variants are associated with total cholesterol, low-density lipoprotein-cholesterol and high-density lipoprotein-cholesterol in normal women. J. Med. Genet..

[31] Weyrich P., Machicao F., Staiger H., Simon P., Thamer C., Machann J., Schick F., Guirguis A., Fritsche A., Stefan N. (2007). Role of AMP-activated protein kinase gamma 3 genetic variability in glucose and lipid metabolism in non-diabetic whites. Diabetologia.

[32] Randrianarisoa E., Lehn-Stefan A., Krier J., Böhm A., Heni M., Hrabě De Angelis M., Fritsche A., Häring H.U., Stefan N., Staiger H. (2020). AMPK Subunits Harbor Largely Nonoverlapping Genetic Determinants for Body Fat Mass, Glucose Metabolism, and Cholesterol Metabolism. J. Clin. Endocrinol. Metab..

